# Detection of COVID-19 case clusters in Québec, May–October 2020

**DOI:** 10.17269/s41997-021-00560-1

**Published:** 2021-08-09

**Authors:** Germain Lebel, Élise Fortin, Ernest Lo, Marie-Claude Boivin, Matthieu Tandonnet, Nathalie Gravel

**Affiliations:** 1 Institut national de santé publique du Québec, Québec and Montréal, Canada; 2 Department of Microbiology, Infectious Diseases and Immunology, University of Montreal, Montréal, QC Canada; 3 Department of Social and Preventive Medicine, Laval University, Québec, QC Canada; 4 Department of Epidemiology, Biostatistics, and Occupational Health, McGill University, Montréal, QC Canada

**Keywords:** COVID-19, Space-time clusters, Disease surveillance, SaTScan, COVID-19, agrégats spatio-temporels, surveillance, SaTScan

## Abstract

**Objectives:**

The Quebec Public Health Institute (INSPQ) was mandated to develop an automated tool for detecting space-time COVID-19 case clusters to assist regional public health authorities in identifying situations that require public health interventions. This article aims to describe the methodology used and to document the main outcomes achieved.

**Methods:**

New COVID-19 cases are supplied by the “Trajectoire de santé publique” information system, geolocated to civic addresses and then aggregated by day and dissemination area. To target community-level clusters, cases identified as residents of congregate living settings are excluded from the cluster detection analysis. Detection is performed using the space-time scan statistic and Poisson statistical model, and implemented in the SaTScan software. Information on detected clusters is disseminated daily via an online interactive mapping interface.

**Results:**

The number of clusters detected tracked with the number of new cases. Slightly more than 4900 statistically significant (*p* ≤ 0.01) space-time clusters were detected over 14 health regions from May to October 2020. The Montréal region was the most affected.

**Conclusion:**

Considering the objective of timely cluster detection, the use of near-real-time health surveillance data of varying quality over time and by region constitutes an acceptable compromise between timeliness and data quality. This tool serves to supplement the epidemiologic investigations carried out by regional public health authorities for purposes of COVID-19 management and prevention.

## Résumé

**Objectifs:**

L’Institut national de santé publique du Québec (INSPQ) a reçu le mandat d’élaborer un outil de détection automatisé des agrégats spatio-temporels des cas de COVID-19 afin d’aider les régions à détecter des situations nécessitant des interventions de santé publique. Cet article vise à décrire la méthodologie utilisée et à présenter les principaux résultats obtenus.

**Méthode:**

Les nouveaux cas de COVID-19 proviennent du Système d’information Trajectoire de santé publique, ils sont géolocalisés à l’adresse civique, puis agrégés par jour et par aire de diffusion. Afin d’isoler la transmission communautaire, les cas identifiés comme résidents d’un milieu de vie fermé sont exclus des analyses de détection des agrégats. La méthode de détection est la statistique de balayage spatio-temporel basée sur le modèle de Poisson et implantée dans le logiciel SaTScan. Les agrégats détectés sont diffusés quotidiennement dans une interface cartographique web interactive.

**Résultats:**

Le nombre d’agrégats détectés varie en fonction du nombre de nouveaux cas. Un peu plus de 4 900 agrégats spatio-temporels statistiquement significatifs (*p* ≤ 0,01) ont été détectés dans 14 régions sociosanitaires entre mai et octobre 2020. La région de Montréal est la plus touchée.

**Conclusion:**

Considérant l’objectif d’une détection d’agrégats en temps opportun, l’utilisation des données de vigie sanitaire en temps quasi réel, dont la qualité est variable dans le temps et selon les régions, constitue un compromis acceptable. Il s’agit d’un outil complémentaire aux enquêtes épidémiologiques menées par les autorités régionales de santé publique dans la gestion et la prévention des impacts populationnels de la COVID-19.

## **Introduction**

In the context of the global health emergency associated with COVID-19, the Quebec Public Health Institute (INSPQ) was mandated to develop an automated tool for detecting space-time clusters of new COVID-19 cases to assist regional public health authorities in identifying situations requiring public health interventions. The purpose of this tool is to identify, in a timely manner, space-time clusters of new COVID-19 cases excluding residents of organized group living environments in the province of Québec, Canada. The aim is to provide an additional instrument that will assist regional public health authorities in identifying areas potentially requiring epidemiologic investigation and supplementary public health protection and prevention measures. Since early May 2020, the results of the cluster detection tool have served to guide public health actions such as the selection of locations for mobile testing sites and the optimization of other preventive interventions.

This article aims to describe the methodology of the tool for detecting space-time clusters of new COVID-19 cases to present the cartographic display of outcomes for the period May–October 2020 and to describe the characteristics of the space-time clusters detected.

## **Methods**

Detection of space-time clusters of COVID-19 cases is performed across the entire province of Québec. As of 2020, Québec had a total population of more than 8.5 million people distributed across an area of 1.5 million km^2^ (the inhabited area, or ecumene, being 110,000 km^2^) (*La Question Démographique (Québec)*, n.d.).

For public health purposes, this territory is divided into 18 health regions. Each region has legal jurisdiction to enforce the *Public Health Act* (Loi sur la santé publique 2001), while the Ministère de la Santé et des Services sociaux du Québec (MSSS) coordinates population health surveillance and protection at the provincial level.

### Data used

New COVID-19 cases are extracted daily from the public health COVID-19 case registry; the provincial case database changed as of July 26^th^. To detect space-time clusters as quickly as possible, positive cases identified by laboratories within the last 10 days, but not yet reported by the regional public health authorities, are added to the cases (effective July 9, 2020). Duplicates were identified and removed using the Québec medical insurance number. Last, the following algorithm determines the corresponding date used based on the availability of the following three dates in order of priority: (1) symptom-onset date; (2) specimen collection date; (3) date reported.

Cases are geolocated using the app iCherche via Open GIS Infrastructure (IGO). Case geolocation is performed using the full home civic addresses of cases. Cases occurring in congregate living settings (e.g., long-term care facilities, private seniors’ residences) are excluded from the analysis. Type of residence is specified in the epidemiologic investigation of new cases. However, this information is not available for cases identified based solely on laboratory results and data entered into the cases database are also frequently incomplete for the newest cases. Consequently, we use the term “community-level clusters” to refer to clusters identified using the detection tool, but acknowledge that these clusters may nevertheless reflect known outbreaks that have occurred in long-term care facilities, in workplaces, or at schools for example. Between May 10 and October 31, 2020, new COVID-19 cases living in congregate living settings accounted for 10% of all new cases.

The geographic unit used for space-time cluster detection is the dissemination area (DA). The DA is the smallest standardized geographic unit for which Canadian census data are available (*Dissemination Area*, n.d.). The population size of DAs for 2020 was estimated using data from the Insured Persons Registry (FIPA) of the Quebec Health Insurance Department (RAMQ). In 2020, the average population of the 13,479 DAs was 630.

### Cluster detection method

Clusters are detected using the space-time scanning statistical method developed by M. Kulldorff (Kulldorff 1997) and incorporated into the free software SaTScan^TM^. The detection method does not take physical barriers (e.g., mountains, rivers) or administrative boundaries into account. As such, a cluster may extend across more than one health region. Clusters may encompass one DA or multiple adjacent DAs.

The use of SaTScan requires the definition of multiple parameters and options, which is a challenge as no gold standard of true clusters exists (Desjardins et al. 2020; Kulldorff 1997, 2001, 2018). A calibration period took place in April–May 2020 during which parameters were defined. Our goal was to identify clusters that were pertinent and useful for guiding preventive interventions by regional public health authorities. Therefore, the size of clusters was limited to that of within neighbourhoods. Also, an attempt was made to limit the number of statistically significant clusters per health region to a manageable level (around 100 in total for the province). Detailed simulations were done in which we varied both the maximum cluster radius and population. As the health regions of Québec vary greatly in size and population density, compromises were made to achieve satisfactory results over all regions.

The minimum number of cases per cluster is 5 in order to limit the detection of clusters occurring within a single household. A cylinder’s maximum radius is reached when the proportion of the population inside the cylinder exceeds 0.25% of the total population of Québec (approximately 20,000) or when the radius equals 15 km. Since the analysis is prospective, the maximum duration of any cluster is 10 days. This window corresponds to twice the mean interval between two linked cases and is intended to capture up to 3 case generations (Du et al. 2020).

The Poisson probability model was used since it considers the at-risk population. Analysis is prospective in order to detect emerging clusters; these clusters are active up to and including the most recent day and consider all new cases within the last 10 days. Only clusters exhibiting a higher-than-expected incidence are subject to detection. Last, the study period was defined to account for all COVID-19 cases since January 1, 2020. The space-time scanning window is cylindrical to include both the spatial and temporal dimensions of clusters. Cylinders are positioned at the centroids of each DA and cover the entire territory of the province of Québec. The radius and duration (height) of each cylinder increase gradually up to the limits defined by the parameter settings in order to assess potential clusters of all possible size and duration within the specified limits. The numbers of new cases observed and predicted inside and outside of each cylinder are used to calculate the relative risk and likelihood ratio for each cylinder. Non-overlapping cylinders with the highest likelihood are retained as clusters. Statistical significance (*p*-value) is established using 999 Monte Carlo simulations (Kulldorff 1997, 2018).

Space-time clusters are detected automatically on a daily basis. A SAS program (SAS Institute Inc. 2012) compiles geolocated cases per day and per DA and generates the files required by SaTScan. Aggregated data are updated daily and made available to users with access to the secure Internet site of the Infocentre de santé publique du Québec. The presence of confidential information limits access of the results only to public health actors identified by regional directors of public health or by the national director of public health (MSSS).

### Characteristics of space-time clusters detected

Two distinct time periods are considered for the descriptive analysis of clusters: the end of the first wave of COVID-19 (May 10–July 11, 2020) (Gosselin et al. 2020) and the start of the second wave (August 23–October 31, 2020). Cluster characteristics include the mean age of cases included, mean radius, and mean relative risk, on a daily basis. Statistically significant clusters (*p* ≤ 0.01) are identified.

## **Results**

An interactive online map and Excel spreadsheets that identify all new cases included in clusters are available on the website of the Infocentre de santé publique. An Excel spreadsheet detailing the daily number of clusters over the past week, by health region, by municipality, and by territory of local community service centres (CLSC) is updated three times a week.

### *Cartographic representation of space-time clusters detected*

**Fig. 1:**
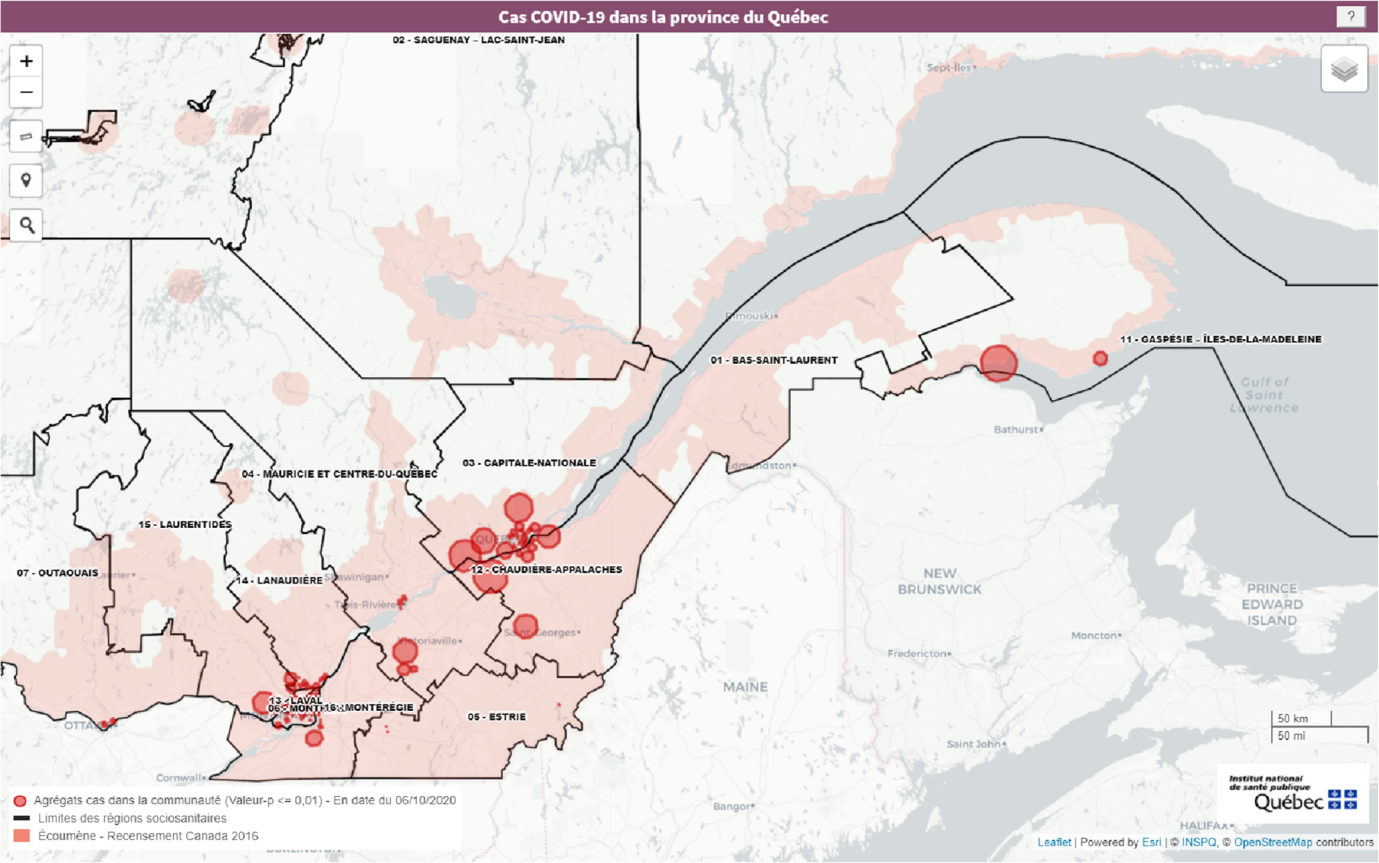
Sample cartographic representation of clusters by health region

**Fig. 2:**
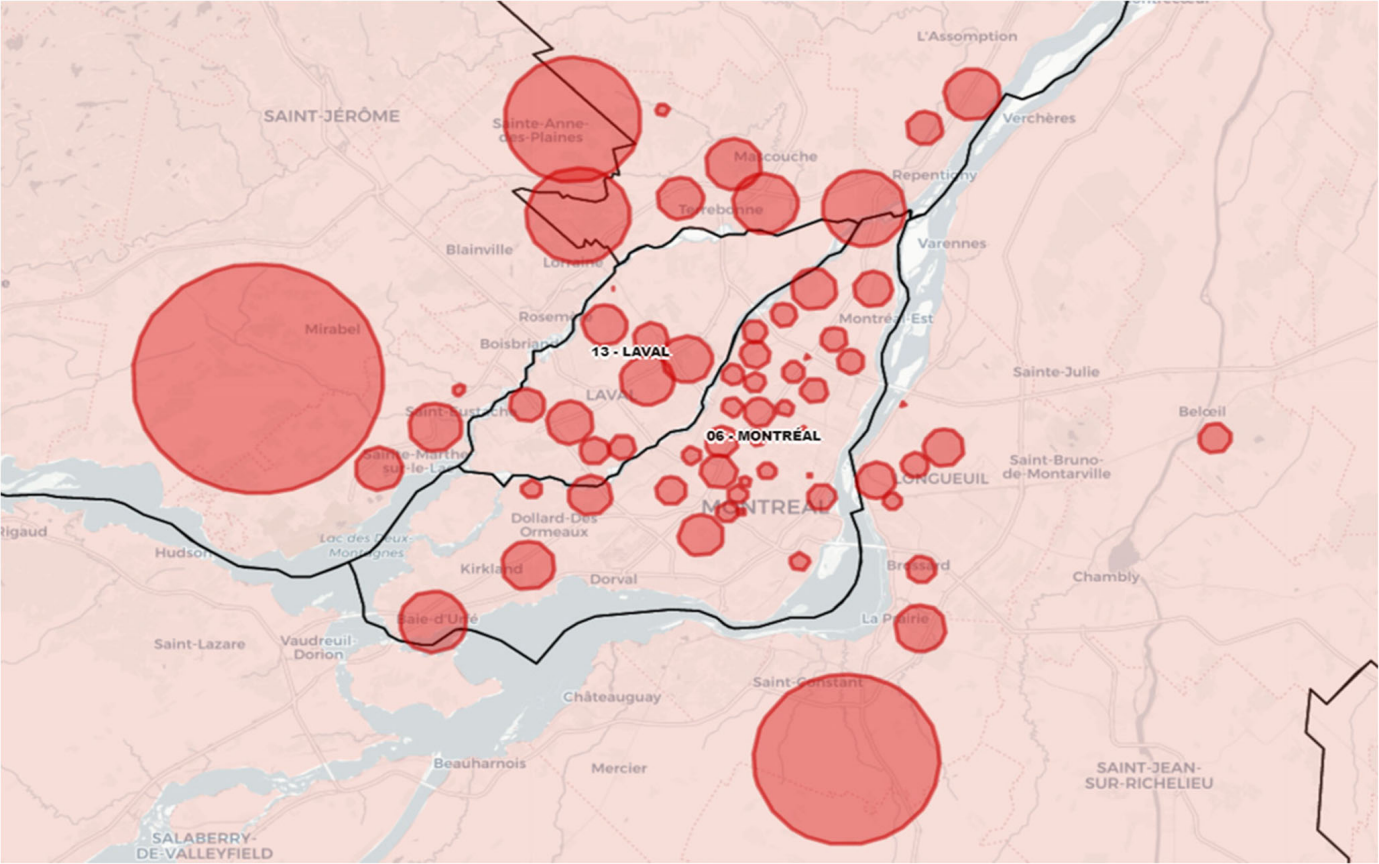
Sample cartographic representation of clusters in the Greater Montréal region

Information on the detected clusters is disseminated via an interactive online map (Leaflet) (Figures 1 and 2). Users can choose to view either all clusters or statistically significant (*p *≤ 0.01) clusters only.

**Fig. 3:**
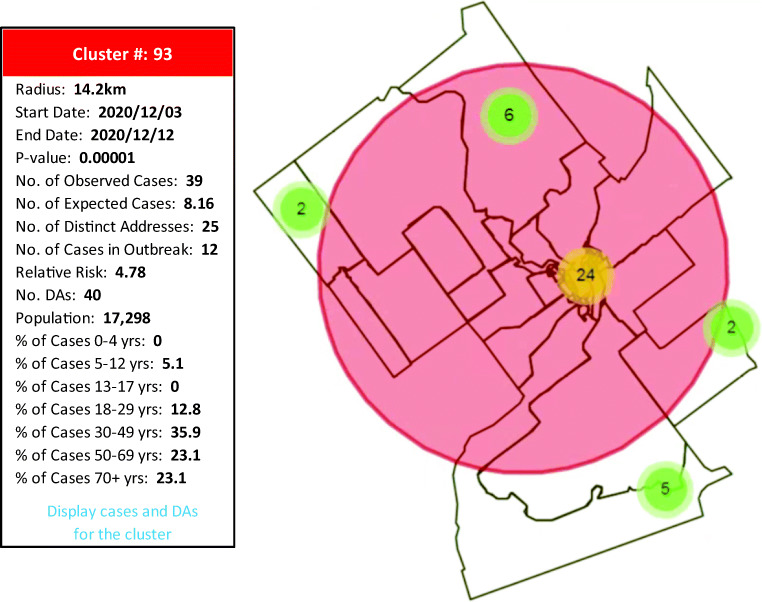
Sample information available per cluster

Users can view the characteristics of the individual clusters detected (e.g., number of cases observed and expected, cluster start and end dates, radius, population, number of DAs) (Figure 3). Various pieces of additional information are also provided to support interpretation at a local level, including number of unique addresses, number of cases in known outbreaks, and frequency distribution of cases by age group (Figure 3). Users can also click a link to view the boundaries of the dissemination areas associated with individual clusters as well as the location of, and information about, each case within each cluster (Figure 3).

### *Characteristics of space-time community-level clusters detected*

#### ***Number of clusters***

**Fig. 4:**
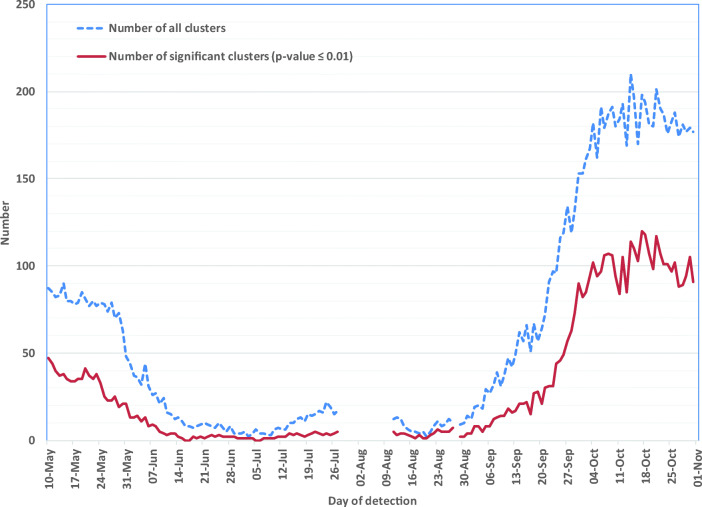
Total space-time community-level clusters of COVID-19 cases detected per day, Québec, May–October 2020. The large gap in the red line (July 28 – August 10) was due to the transition between COVID-19 case information systems, during which geolocalized case data were not available. The small gap that occurs on August 28 was due to the absence of geolocalized case data for that day

Figure 4 illustrates the total number of space-time clusters detected province-wide. The number of clusters detected followed variations in the daily frequency of new cases. During May and September, for example, more than 70 clusters per day occurred on average. Between July 27 and August 10, cluster detection was not possible due to the switchover of the COVID-19 case information system (Gosselin et al. 2020). The number of clusters declined between May 10 and mid-June toward the end of the first COVID-19 wave. The number of clusters then remained low, at an average of 9 clusters per day, from mid-June through late July. In early September, the number of clusters increased substantially before reaching a plateau in early October, indicating the advent of the second wave of COVID-19 in the province. More than 180 clusters per day on average were identified during the month of October (Figure 4).

**Fig. 5:**
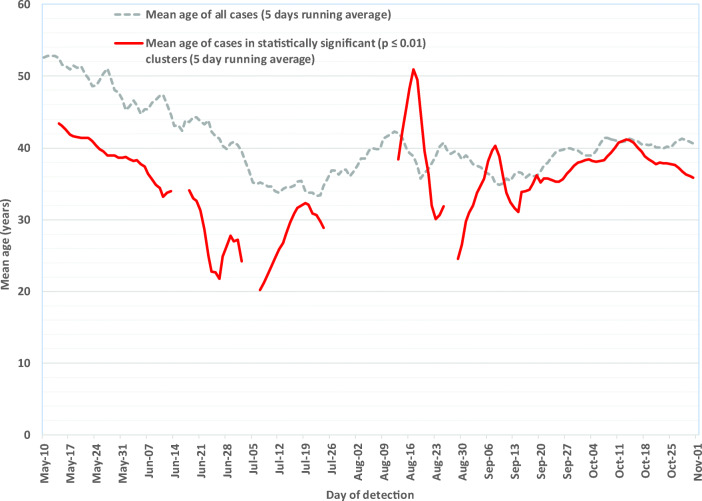
Mean age of COVID-19 cases (all cases combined and cases included in significant community-level clusters), Québec, May–October 2020. The first two small gaps in the red line (June 16–17 and July 5–6) are due to the absence of any statistically significant clusters during those periods. The large gap in the red line (July 28 – August 10) was due to the transition between COVID-19 case information systems, during which geolocalized case data were not available. The small gap that occurs on August 28 was due to the absence of geolocalized case data for that day

**Fig. 6:**
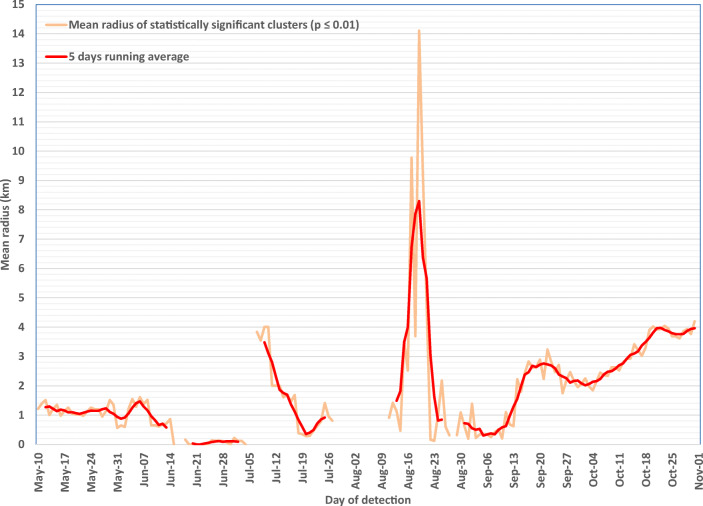
Mean radius of statistically significant community-level clusters detected by day, May–October 2020. The first two small gaps (June 16–17 and July 5–6) are due to the absence of any statistically significant clusters during those periods. The large gap (July 28 – August 10) was due to the transition between COVID-19 case information systems, during which geolocalized case data were not available. The small gap that occurs on August 28 was due to the absence of geolocalized case data for that day

Table 1 enumerates the daily number of statistically significant clusters detected per region, per period. The Montréal region was the region most affected throughout the study period. Montréal accounted for 64% of all clusters detected between May 10 and July 11 (end of first wave). The proportions of statistically significant clusters in three other regions (Laval, Montérégie and Lanaudière) were higher during the first wave than during the second wave (late August). In all other regions, the majority of statistically significant clusters were observed early in the second wave, between July 12 and October 31.

**Table 1:** Number of statistically significant (*p* ≤ 0.01) community-level clusters detected per health region, per period

**Health region**	**May 10 to October 31, 2020**		**May 10 to July 11, 2020**		**July 12 to October 31, 2020**	
	***n***	**%**	***n***	**%**	***n***	**%**
Montréal	1875	38.2%	557	64.2%	1318	32.6%
Capitale-Nationale	690	14.1%	3	0.3%	687	17.0%
Montérégie	547	11.1%	94	10.8%	453	11.2%
Laval	472	9.6%	142	16.4%	330	8.2%
Chaudière-Appalaches	379	7.7%		0.0%	379	9.4%
Lanaudière	362	7.4%	90	10.4%	272	6.7%
Mauricie and Centre-du-Québec	271	5.5%	8	0.9%	263	6.5%
Estrie	159	3.2%	3	0.3%	156	3.9%
Laurentides	135	2.8%	18	2.1%	117	2.9%
Outaouais	92	1.9%		0.0%	92	2.3%
Gaspésie – Îles-de-la-Madeleine	86	1.8%		0.0%	86	2.1%
Saguenay – Lac-Saint-Jean	59	1.2%		0.0%	59	1.5%
Bas-Saint-Laurent	47	1.0%		0.0%	47	1.2%
Abitibi-Témiscamingue	1	0.0%		0.0%	1	0.0%
**Province of Québec**	**4909**	**100%**	**867**	**100%**	**4042**	**100%**

**Fig. 7:**
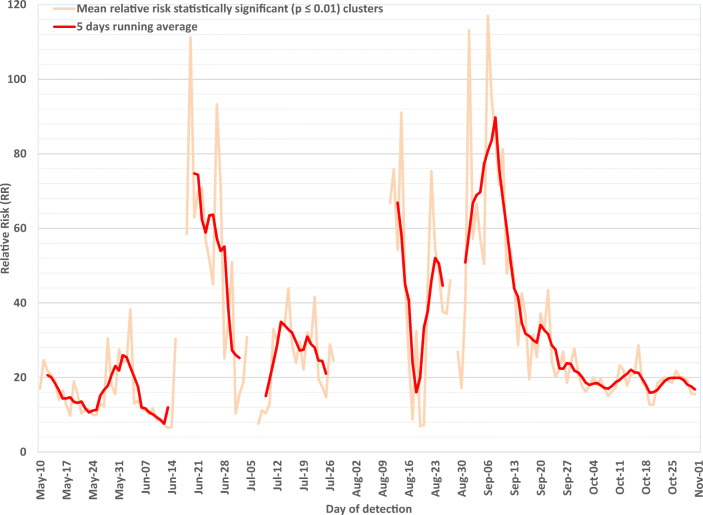
Mean relative risk of statistically significant community-level clusters, Québec, May–October 2020. The first two small gaps (June 16–17 and July 5–6) are due to the absence of any statistically significant clusters during those periods. The large gap (July 28 – August 10) was due to the transition between COVID-19 case information systems, during which geolocalized case data were not available. The small gap that occurs on August 28 was due to the absence of geolocalized case data for that day

#### ***Mean age of new cases in statistically significant clusters***

The mean age of cases occurring in statistically significant clusters reveals trends similar to those associated with the mean age of all COVID-19 cases (Figure 5). The mean age of cases included in statistically significant community-level clusters was 33.5 years for the period between May 10 and July 11, 2020, versus 44.5 years for all cases (all cases include long-term care facilities and nursing homes) during the same period. During summer, the number of cases decreased, and the mean case age in the detected clusters fluctuated substantially. At the start of the second wave, or beginning in late August, the mean age of cases in statistically significant clusters (36 years) was closer to the mean age of all cases (39 years).

#### ***Mean radius of statistically significant clusters***

A cluster’s mean radius defines the size of its cylinder base and, consequently, the cluster’s geographic range. Mean radius values varied more widely when the number of clusters detected was low (e.g., during August 17–21, Figure 6). Between May 10 and July 11, the mean radius of statistically significant clusters was small (temporal average: 0.98 km, range: 0–4.0 km) (Figure 6). Between August 23 and October 31, at the start of the second wave, the mean radius of significant clusters detected was 2.20 km (range: 0.12–4.2 km). An upward trend in the daily mean radius was observed during October (Figure 6).

#### ***Mean relative risk of statistically significant clusters***

Mean relative risk per day for statistically significant clusters is depicted in Figure 7. Relative risk is equal to the observed to expected incidence rate ratio within a cluster, where the expected incidence rate corresponds to the average incidence rate over the entire province and study period. Trends in mean relative risk followed those of the total number of daily cases. Between early May and July 11, the mean relative risk of significant clusters was 21.1 (range: 5.6–111.3). Substantial fluctuations were then observed through mid-September when the number of clusters was lower. From mid-September until October 31, the mean relative risk was 8.7 (range: 6.6–20.9). It became lower and relatively more stable during the month of October.

## **Discussion**

Our COVID-19 cluster detection tool has been available to regional public health stakeholders since early May 2020. This SaTScan-based, automated tool provides timely and useful signals on a daily basis via an online mapping interface. These data can be used to quickly identify emerging groupings of new COVID-19 cases that may correspond to outbreaks of community-level transmission. For regional public health authorities, these types of space-time clusters are more difficult to identify than outbreaks within families or in congregate living settings. Since the tool uses cases’ home addresses, detection does not specifically target the school, daycare, healthcare, or work environments. However, public health authorities track and investigate outbreaks specific to these environments using other tools and methods. Through our interactions with the regional authorities, various uses of the tool were brought to our attention. It has been used to determine where mobile screening clinics could be located or where information sheets should be distributed. It was sometimes used to check whether outbreaks had been missed or if cases had been missed in the investigation of already known outbreaks. Finally, in periods of very high incidence of the disease, the tool became a more efficient way to detect hot spots in a timely manner.

Clusters are detected at the spatial resolution of very small geographic units in the form of DAs. This precise geolocation supports highly accurate location of clusters that will assist regional public health authorities in more effectively targeting preventive interventions. In addition to cluster characteristics, the online map has additional features that support daily analysis including the precise case location (by home address), case frequency distribution by age group, number of cases that correspond to known outbreaks, and number of unique civic addresses in each cluster. Using the Excel spreadsheets, users have the option to sort clusters by *p*-value to identify the most statistically significant clusters to prioritize epidemiologic investigations by regional public health authorities. These features have been added to enhance the outcomes supplied by SaTScan. This information is useful to regional public health authorities seeking to better understand the nature of clusters and identify priority geographic areas for testing, prevention, and public health protection activities.

The space-time scanning method has been used for some time to analyze the incidence of various infectious diseases in near-real-time in both Montréal (Cadieux et al. 2020) and the United States (Greene et al. 2016; Hughes and Gorton 2013). This method is also already widely used for COVID-19 (Amin et al. 2020; Andrade et al. 2020; Clinique 2020; Desjardins et al. 2020; Greene et al. 2020; Hohl et al. 2020). Other methodologies are also available, for example, those based on Bayesian models, but these were not considered due to the urgency of the situation and our team’s skills. In one 2010 publication, the use of SaTScan was recommended over three other applications considered for space-time cluster detection (Robertson and Nelson 2010). Other statistical models in SaTScan, such as the space-time permutation method, were tested during the calibration phase. The Poisson model appeared most appropriate since the population sizes for 2020 were available by DA and this method can be used to estimate risk.

The number of clusters detected appears to vary with both the frequency of new cases and the epidemiologic characteristics of cases. For example, the first wave in Québec was characterized by a high number of cases in seniors’ residences (Gosselin et al. 2020). As a result, the number of community-level clusters is much lower during that period than in the beginning of the second wave. The start of the second wave appears to be characterized by a much higher extent of community transmission as well as numerous outbreaks in workplaces and schools. The differing characteristics of the two COVID-19 waves in Québec could explain the fact that the mean case age in statistically significant clusters was lower at the start of the second wave as compared with the first wave: long-term care facilities, nursing homes, and the elderly in general were hit particularly hard during the first wave while the virus had spread to all age groups during the second wave. Information on the age distribution of cases within clusters is an information that helped regional public health authorities in their intervention planning and we could see that the tool’s results followed the expected time trends.

During the start of the second wave, COVID-19 had spread more widely out of the Montréal area. The statistically significant clusters detected during this phase of the pandemic occurred more frequently in less densely populated regions (Table 1) in contrast with the end of the first wave. The mean radius of clusters was also higher during the start of the second wave. This reflects the occurrence of clusters in less urban areas that have lower density population and case counts. In these areas, clusters likely consist of relatively sparsely distributed cases extending over larger ranges; also, the specified population limit of 20,000 permits clusters with higher radii than in denser urban areas. The observed decline in mean cluster relative risk could also be explained by an increased province-wide global risk level or incidence.

There are certain limitations inherent to the use of SaTScan. Selection of parameters and analysis options represented a first challenge. Given the lack of historical data on COVID-19, there was no way to guarantee the accuracy or pertinence of the space-time clusters detected under a chosen parameter setting. The complex and heterogeneous population across the province of Québec also had to be considered. Some regions consist strictly of a densely populated urban environment, while other regions have both urban cores and rural areas and still others are almost predominantly rural. Last, the specification of parameters and calibration phase were carried out during the first wave, when community transmission was relatively low, with a large proportion of cases occurring among residents of long-term care homes and healthcare workers.

Data quality poses another challenge. The use of health surveillance data available in near-real-time has certain limits. Data completeness and quality remain the Achilles’ heel in the daily detection of space-time clusters. Home addresses are frequently incomplete or, in some cases, missing in the most recently acquired data, thereby preventing their consideration during cluster detection. The fact that a case resides in an organized group living environment is also not always indicated. For example, cluster detection is performed using the most recent data available, which includes laboratory-positive cases that have not yet been reported to regional public health authorities; the residential environment is missing in these cases. Overall, the availability of dates and information on place of residence varies from day to day and is poorer during high-transmission periods. The use of these sometimes incomplete data is considered, however, an acceptable compromise in light of the objective of timely cluster detection.

## **Conclusion and perspectives**

The space-time cluster detection tool aims to identify unusual aggregations (i.e., clusters) of new COVID-19 cases, excluding residents of congregate living settings, in the province of Québec. This tool supplements existing processes put in place by regional public health authorities for the epidemiologic investigation of COVID-19 outbreaks. The tool’s outcomes are used by several regional public health departments as part of their pandemic management. The performance of the detection tool depends on parameters defined in advance as well as on data availability, validity, and quality. Since the tool enables the automated detection of clusters, it appears to be more useful to public health authorities when the daily number of new cases is high (strong transmission) and when the capacity to conduct exhaustive epidemiologic investigations is more limited.

Evaluation of tool performance in regard to detection sensitivity and timeliness is ongoing. As we seek to continually improve the tool and its utility to public health actors, we are currently exploring alternate parameter settings, such as the use of a shorter, rolling study period synchronized to the most recent data acquisition date.

**Contributions to knowledge**

What does this study add to existing knowledge?

- This study describes the near real-time COVID-19 cluster detection tool developed in the province of Québec.
- The methodology is described as well as outputs which include an interactive online map and detailed information on each cluster.
- Characteristics of detected clusters from the end of the first wave of the pandemic (May 10 – July 11) to the start of the second wave (August 23 – October 31) are described.

Implications for public health interventions, practice or policy

- The Québec COVID-19 cluster detection tool is currently being used by regional public health authorities in Québec to guide protection and prevention measures such as the allocation of mobile testing sites and identification of areas for priority testing.
- The tool appears to be especially useful during periods of high transmission.
- The development, methodology and outcomes of the Québec cluster detection tool may help to guide the development of similar tools by public health authorities in other countries or regions.
